# Anxiety, depression, stress, fear and social support during COVID-19 pandemic among Jordanian healthcare workers

**DOI:** 10.1371/journal.pone.0247679

**Published:** 2021-03-12

**Authors:** Eman Alnazly, Omar M. Khraisat, Ahmad M. Al-Bashaireh, Christine L. Bryant

**Affiliations:** 1 Department of Primary Care Nursing, Faculty of Nursing, Al-Ahliyya Amman University, Amman, Jordan; 2 HSHS St John’s Hospital, Springfield, Illinois, United States of America; Universitat d’Alacante, SPAIN

## Abstract

The emergence of Coronavirus disease 2019 (COVID-19) has affected health-care workers’ psychological and mental health. Few studies have been conducted examining the psychological effect of COVID-19 on health-care worker psychological health in Jordan. Therefore, the present study aims to assess the respective levels of fear, anxiety, depression, stress, social support, and the associated factors, experienced by Jordanian health-care workers during the COVID-19 Pandemic. This study adopted a cross-sectional, correlational design to collect data from 365 health-care workers in Amman, Jordan, from August 16th to 23rd, 2020. Along with collecting sociodemographic characteristics, the Fear of COVID-19 Scale, the Depression, Anxiety, Stress Scale, and the Multidimensional Scale of Perceived Social Support electronically administered to participants. The majority of the participants (69.3%) were registered nurses. The mean overall score for the Fear of COVID-19 scale was 23.64 (SD + 6.85) which again exceeded the mid-point for the total score range (21), indicating elevated level fear of the COVID-19 pandemic. Participants had displayed extremely severe depression 40%, extremely severe anxiety 60%, and 35% severely distressed. Scores for depression (21.30 ± 10.86), anxiety (20.37 ± 10.80), stress (23.33 ± 10.87) were also high. Factors determined to be associated with psychological distress were being male, married, aged 40 years and older, and having more clinical experience. Assessment of social support indicated moderate-to-high levels of perceived support for all dimensions (significant other: 5.17 ± 1.28, family: 5.03 ± 1.30, friends: 5.05 ± 1.30). Weak significant correlations were found between social support and the other study variables (r < 0.22), indicating a weak association with fear, depression, anxiety, and stress, respectively. Overall, Jordanian health-care workers sample reported fear, depression, anxiety, and stress. The associated factors were being male, married, aged 40 years and older, and having more clinical experience. Regarding social support, participants primarily relied on support from their families, followed by support from friends.

## Introduction

On March 11th, 2020, the World Health Organization (WHO) declared that the coronavirus disease 2019 (COVID-19) outbreak had become a pandemic [1]. In December 2019, reports emerged from China regarding the initial detection of SARS-COV-2 as the source of the pneumonia outbreak of COVID-19 [2]. On January 26th,2020 the Jordanian National Epidemic Committee and the Jordanian Ministry of Health had met to put a plan in place to manage the pandemic. The recommendations had included the designation of several hospitals as treatment centers for prospective patients with COVID-19 and established protocols to prevent the spread of the country’s infectious disease even before the first case of COVID-19 was reported [3]. The Jordanian Ministry of Health had followed the Epidemic committee’s recommendations and opened five hospitals located in different areas around the country designated for treating patients with COVID-19. Ministry of Health had equipped these hospitals with ventilators, personal protective equipment (PPE), including disposable gowns, masks, gloves, and face-shields, and trained infectious disease medical staff [3]. Besides, the Jordanian Ministry of health advised all health-care workers from different sectors to wear PPE and implemented quarantine policies [3]. The first case of COVID-19 in Jordan was reported on March 2nd [3], and on March 15th, the government closed the country’s borders, suspended schools, banned public gatherings, and issued a stay-at-home order [3]. On March 17th, after a case of COVID-19 was traced to a wedding in north Jordan, the government implemented a curfew [4]. On March 25th, the government lifted the curfew partially and allowed essential services and schools to remain closed. Ministry of Health mandated social distancing, masks in public, and the self-quarantining of asymptomatic positive persons. By the end of April, there were 451 registered cases and 8 deaths [4].

By mid-August, the COVID-19 situation in Jordan began to worsen, with the recording of 20–30 cases per day and toward the end of August, the daily cases were 30–40 [4]. This quickly escalated to several hundred and then to several thousand cases a day, most likely due to a lack of public compliance with recommendations; consequently, the government imposed stricter safety measures and penalties for non-adherence [4].

In Jordan, during the month of August, there were 2,034 confirmed cases (including 50 health-care workers), 456 people receiving treatment, 1,508 recovered cases, and 15 deaths [4]. On the global level, by August 15^th^, 300,000 health-care workers worldwide had contracted COVID-19, and 2,500 had died [5]. Further, over 1.8 million new COVID-19 cases and 38,000 new deaths were reported worldwide during August; this meant a cumulative total of 25 million cases and 800,000 deaths since the beginning of the outbreak [6]. During the month of November, a total of 817 cases had been recorded among nurses, representing 5.5% of health-care workers, and 26 COVID-19-related deaths had been recorded among physicians [7]. It should be noted that the figures above regarding case numbers among health-care workers almost certainly do not reflect the actual number of cases among health-care workers, as some infected people exhibit mild symptoms or no symptoms, meaning they are unlikely to be tested [8]. The COVID-19 has significant negative impacts on health-care workers’ psychological health, fostering issues such as anxiety, depression, and sleep disturbance [9]. This indicates the necessity of providing psychological support for health-care workers, such as by implementing occupational health surveillance programs that train and educate health-care workers in terms of their ability to address the infectious disease and associated psychological distress [9].

Moral injuries are a form of psychological distress that result from performing an action that contradicts one’s own moral and ethical code; such incidents can produce emotional guilt, shame, and anger [10]. These symptoms can contribute to mental-health difficulties, which can lead to either psychological injury or psychological growth [10]. Whether an individual experiences the former or latter consequence is likely to be influenced by how he/she is supported before, during, and after the incident [11]. Health-care workers have been found to experience moral injuries, as well as isolation, and at risk for occupational injuries, and life-threatening situations [9]. Occupational defines as injuries relate to any disease caused by any biological agent that can be experienced while working or while commuting to work [12].

As a result of the pandemic, rapid spread and the associated increased mortality rate, the pandemic has caused public-health issues worldwide; further, the stress people experience in response to this situation has also had a severe negative effect [13]. Regarding health-care workers, COVID‐19 has caused issues such as high health-care demands, increased patient mortality, emotional and physical stress, and rationing of health-care supplies [14]. Further, rapid increases in the number of suspected and confirmed positive cases, low supplies of PPE, overwhelming work-loads, widespread media coverage of the pandemic, perceived inadequate organizational support, and an increased risk of contracting the disease and transmitting it to one’s own family have also caused psychological distress among health-care workers [14–16]. It is essential to consider both the psychological and physiological influence of the pandemic on health-care workers. Failure to assess and address psychological responses to pandemic-associated stressors can negatively impact health-care workers’ physiological and psychological functioning [13]. Notably, during pandemics, health-care workers who provide care to patients are among the populations most likely to experience psychological distress, including depression and anxiety [14–17].

Previous studies of COVID-19 pandemics have revealed that the psychological effects of infectious disease outbreaks can last long after the event, negatively impacting psychological well-being [18] and causing post-traumatic stress disorder, depression, and stress among health-care workers [19, 20]. In the context of the pandemic crisis, health-care workers are expected to deal with patients’ traumatic experiences and the unexpected loss of friends, family, and colleagues. As a result, health-care workers are affected by psychological distress, including depression, anxiety, and stress [21]. Batra et al. [22] conducted a meta-analysis to provide new evidence related to COVID-19 impact on health-care workers’ psychological well-being. Among the main factors identified as causal in psychological distress are anxiety, depression, stress, post-traumatic stress syndrome, insomnia, psychological distress, and burnout. Higher anxiety and depression levels were more prevalent among females than males and nurses compared to doctors and front-line workers compared to second-line health-care workers [22].

There are four categories of social support: "emotional," "appraisal," "informational," and "instrumental" [23]. Social networks include an individual’s family, friends, neighbors, and other close significant persons [23]. For health-care workers, social support reduces occupational stress and prevents common psychological distress and psychiatric symptoms; however, coworker support is also significant for health-care workers, as it impacts self-efficacy and professional efficacy [24]. Notably, negative social support is associated with stress and anxiety among medical staff [15].

COVID-19 is an infectious disease that has affected virtually every nation in the world. Research has currently focused on addressing the general population’s well-being with little attention being directed toward health-care workers’ psychological distress. Therefore, the present study aimed to assess the fear, depression, anxiety, stress, social support, and the associated factors among Jordanian health-care workers during the COVID-19 pandemic. Also, we aimed to investigate the impact of sociodemographic characteristics on these variables.

Through this analysis, we determined that health-care workers in Jordan have high levels of depression, anxiety, stress, and fear of COVID-19, but that they also perceive high levels of social support.

## Methods

### Study design and participants

This quantitative study featured a cross-sectional, descriptive, and correlational design. The participants were 365 health-care workers from Amman, Jordan, who completed an online questionnaire distributed through Google Forms between August 16th and August 23rd, 2020 when COVID-19 situation in Jordan began to worsen, with the recording of 20–30 cases per day and this quickly escalated to several hundred and then to several thousand cases a day. However, the number of cases that required hospitalization was low. Individuals were approached for participation through social-media applications, text messaging, and emails.

The online Raosoft sample size calculation methodology was used in our study [25]. According to this method a minimum of 378 participants is needed; given that the margin of error alpha (α) = 0.05, the confidence level is = 95%, total population = 21,033 [26], and the response of distribution = 50%. The sample size was also calculated using Krejcie and Morgan method, which provides a similar sample size [27]. Our study was able to recruit a close number of 365 participants.

### Participant recruitment

For initial recruitment, the present researchers contacted 24 health-care workers (Registered Nurses, Pharmacists, physicians, radiologist), who were known to the researchers. Through individual phone calls, the researchers informed these coworkers of the purpose and procedure of the study. The researchers then asked the group if they knew of any other health-care workers who met the inclusion criteria (see below), and if they could invite them to participate in this study. An informational document that provided details regarding the survey (i.e., the title of the study, the purpose and significance of the study, privacy information, and researchers’ email addresses and phone numbers) was distributed to prospective participants. The group forwarded the informational document to other health-care workers through email, text message, or social media. Health-care workers who agreed to participate were contacted by a member of the research team through email or text message. Any questions these prospective participants had regarding the study were answered. A URL linking to the consent form was sent to each individual who agreed to participate, and consent to participate was confirmed through electronic signature (i.e., the ticking of a box on the form). After consent was received, a URL for the Google Forms questionnaire was sent to the participants by text message or email. The researchers emailed the URL to 510 health-care workers, returned 365 (72%) responses.

### Inclusion and exclusion criteria

Inclusion criteria for participation were: 1) being a health-care worker, 2) residing in Amman/Jordan, and 3) providing care for patients at the time of the survey. The exclusion criterion was not working the week prior to the data-collection period.

### The e-survey

The survey was administered online, and the Checklist for Reporting Results of Internet E-Surveys (commonly known as “CHERRIES”) [28] was used to report the results. The online questionnaire was developed using Google Forms. Google Forms represents a method of quickly gathering participants’ responses online. The survey answers were automatically collected in an EXCEL spreadsheet that was imported into SPSS for data analysis. To determine the practicability of the questionnaire, the constituent instruments were pilot-tested beforehand on a group of 30 health-care workers; these individuals were excluded from the main study.

### The research instruments

#### Sociodemographic characteristics and health-related variables

Participants’ socio-demographic characteristics, including gender, age, education level, marital status, profession, work type, and clinical experience, were collected.

#### The fear of COVID-19 scale

The participants were asked to report their level of fear regarding the COVID-19 Pandemic. The Fear of COVID-19 Scale (FCV-19S) is a seven-item scale designed to measure fear of COVID-19 among the general population [29]. Answers are given using a five-point scale (1 = “strongly disagree,” 2 = “disagree,” 3 = “neither agree nor disagree,” 4 = “agree,” and “5 = strongly agree”). The scores for all seven items are summed to obtain the total score; thus, the range for the total score is 7–35. Higher scores indicate greater fear of COVID-19. The scale has acceptable concurrent validity when compared with the Hospital Anxiety and Depression Scale and the Perceived Vulnerability to Disease Scale; further, the developers determined that the Cronbach’s alpha value for the FCV-19S is 0.82, and that its test-retest reliability is 0.72 [29]. For this study, the Cronbach’s alpha value was 0.91.

#### Depression, anxiety, stress scale

The Depression, Anxiety, Stress Scale (DASS) is designed to measure respondents’ depression, anxiety, and stress, respectively, over the past seven days [30]. The scale comprises three self-reported subscales, and has a total of 42 items. Each subscale comprises 14 items. Items are rated using a four-point Likert scale ranging from 0 to 3 (0 = “not at all,” 1 = “to a considerable degree, or some of the time,” 2 = “most of the time,” 3 = “all the time”).

The respective scores for depression, anxiety, and stress were calculated by totaling the scores for the respective associated items, and the severity rating index was used to determine the respondent’s status in each regard. The severity rating index for each DASS subscale as follow (depression was comprising normal (0–9), mild (10–13), moderate (14–20), severe (21–27), and extremely severe (28+). Anxiety scoring comprising normal (0–7), mild (8–9), moderate (10–14), severe (15–19), extremely severe 20+. Stress scoring comprising normal (0–14), mild (15–18), moderate (19–25), sever (26–33), extremely severe (34+). In the original study, the Cronbach’s alpha values for depression, anxiety, and stress were 0.91, 0.84, and 90, respectively [30]. For this study, the Cronbach’s alpha values for depression, anxiety, and stress were 0.95, 0.94, and 0.96, respectively.

#### Multidimensional scale of perceived social support

The Multidimensional Scale of Perceived Social Support (MSPSS) is designed to determine respondents’ perceptions regarding the adequacy of the support they receive from family, friends, and significant others. The MSPSS [31] is a 12-item self-administered scale, and responses are given using a seven-point Likert scale (1 = “very strongly disagree,” 2 = “strongly disagree,” 3 = “mildly disagree,” 4 = “neutral,” 5 = “mildly agree,” 6 = “strongly agree,” 7 = “very strongly agree”). The scale comprises three subscales: family, friends, and significant others. For each subscale, the mean score is determined by summing the scores for each associated item and dividing the result by 4. The total score is determined by summing the scores for each of the 12 items. For the original study, the Cronbach’s alpha values were 0.91, 0.87, and 0.85 for the significant others, family, and friends subscales, respectively. The reliability of the total scale was 0.88. Further, the test-retest reliability after 2–3 months was 0.91, 0.85, and 0.75 for the significant others, family, and friends subscales, respectively, and 0.85 for the overall scale [31]. For the present study, the Cronbach’s alpha values were 0.89, 0.86, and 0.87 for the significant other, family, and friends subscales, respectively.

### Scale administration

The validity of three questionnaires was established using a panel of six experts to ensure the validity of the questionnaires. The validity checked in terms of the survey questions measures what they were intended to measure (face validity), the survey contains questions that covered all aspects of the construct being measured (construct validity), and the extent to which a constructed measure may relate to or predict any outcome for another measure (criterion validity) [32]. The six experts are faculty members of PhD holders with a specialty in mental health, medical-surgical, and community. All experts agreed that the questionnaires were valid.

The three scales were administered in English. The instruments were pilot-tested on 30 health-care workers who were known to the researchers; these individuals were excluded from the main study. The Cronbach’s alpha values obtained through the pilot test were as follows: FCV-19S = 0.86, DASS = 0.90, and MSPSS = 0.84. The test-retest reliability for the same group was as follows: FCV-19S = 0.88, DASS = 0.82, and MSPSS = 0.80.

### Ethical considerations

This study was performed in accordance with the Declaration of Helsinki, and approval was obtained from the Human Subjects Review Board of Al Ahliyya Amman University (ID number: 2020-2019/14/5) prior to the data collection. Written informed consent was obtained from all participants. The data were stored on a personal computer to which only the main author had access.

### Statistical analyses

Data were entered and analyzed using SPSS software (IBM, SPSS Statistics, Version 24). Initially, the data were checked for missing data and outliers. There was no missing data because, on e-survey, we had a star on each question that participants could not move to the next question without answering the previous question. The outliers were screened through visual assessment for scattered plot diagrams, which revealed no outliers. Box Plot and histogram were used to check the normality, as well as the linearity was checked by Pearson correlation, and homogeneity was checked by The Levene’s test.

Descriptive statistics were used, including frequencies (n), percentages (%), means, standard deviations (SDs), medians, and interquartile ranges (IQRs). Variations between sub-categories of demographic variables were checked using chi-square tests. Inferential statistics approaches were used to identify differences in demographic variables; these approaches included independent samples t-tests and variations across demographic sub-groups. Further, Pearson’s correlation coefficient was used to determine the relationships between variables and to establish the inter-correlation matrix. To lower the risk of type I errors, the statistical significance level was set at p < 0.05.

## Results

The participants’ sociodemographic characteristics are presented in Table 1. Participants were distributed over a range of demographic subgroups. Approximately 55% of the participants were women, and most were aged below 50 years (77.8%) and were married (57.5%). The median family size was three members. Most participants were registered nurses (63.0%), held a baccalaureate degree (69.3%), and provided direct care to patients (75.9%). Over 65.0% of the participants had over 10 years of clinical experience. Questions regarding the COVID-19 Pandemic revealed that most of the participants (62.7%) had never provided direct care for patients who had tested positive for COVID-19. However, most of the participants (73.2%) reported receiving support from work administrators during the COVID-19 Pandemic, and 58.4% reported high adherence to the stay-at-home regulations.

**Table 1:** Participants’ sociodemographic characteristics (N = 365).

Variable		n (%)	Chi-square
			*p* value
**Gender,** n (%)	Male	162 (44.4)	0.032
	Female	203 (55.6%)	
**Age,** n (%)	20–29	117 (32.1%)	< 0.001
	30–39	79 (21.6%)	
	40–49	88 (24.1%)	
	50–59	61 (16.7%)	
	≥ 60	20 (5.5%)	
**Marital status**, n (%)	Single	155 (42.5%)	0.004
	Married	210 (57.5%)	
**Family size**, median (IQR)		3 (2–4)	
**Profession**, n (%)	Registered Nurse	230 (63.0%)	< 0.001
	Physician	36 (9.9%)	
	Nurse aid	34 (9.3%)	
	Radiologist	35 (9.6%)	
	Pharmacist	30 (8.2%)	
**Education**, n (%)	Associate	12 (3.3%)	< 0.001
	Bachelor’s	253 (69.3%)	
	Postgraduate	100 (27.4%)	
**Type of work**, n (%)	Direct contact with patients	277 (75.9%)	< 0.001
	Administrator	67 (18.4%)	
	Other	21 (5.8%)	
**Clinical experience (years)**, n (%)	< 10	119 (32.6%)	0.001
	10–19	152 (41.6%)	
	≥ 20	94 (25.8%)	
**Provided care for patients who were COVID-19-positive,** n (%)	Yes	136 (37.3%)	< 0.001
	No	229 (62.7%)	
*Received support from work administrators during the pandemic, n (%).	Yes	267 (73.2%)	< 0.001
	No	98 (26.8%)	
1. Regular communication. 2. Felt that staff well-being was being prioritized (e.g., through provision of a safe working environment, sufficient staff, PPE). 3. Felt that staff were monitored for symptoms of mental distress, burnout, fatigue, and unrest, and that home-related responsibilities (e.g., baby-sitting) were considered. 4. Felt that staff were kept informed and that efforts were made to raise awareness of the pandemic. 5. The organization established an employee health center.			
**Followed stay-at-home policies,** n (%)	Yes	213 (58.4%)	0.001
	No	152 (41.6%)	
**Took vacation days in the last two weeks, during the pandemic,** n (%)	Yes	150 (41.1%)	< 0.001
	No	215 (58,9%)	

* For each participant, mean scores for each of these items were calculated. If, when the mean scores for each item were summed, the overall score was below 2.5, the participant was considered to have received insufficient organizational support (representing “no”); if the score was above 2.5, the participant was considered to have received satisfactory organizational support (“yes”).

IQR: inter-quartile range; PPE: personal protective equipment.

### Assessment of fear of COVID-19

**Fig 1:**
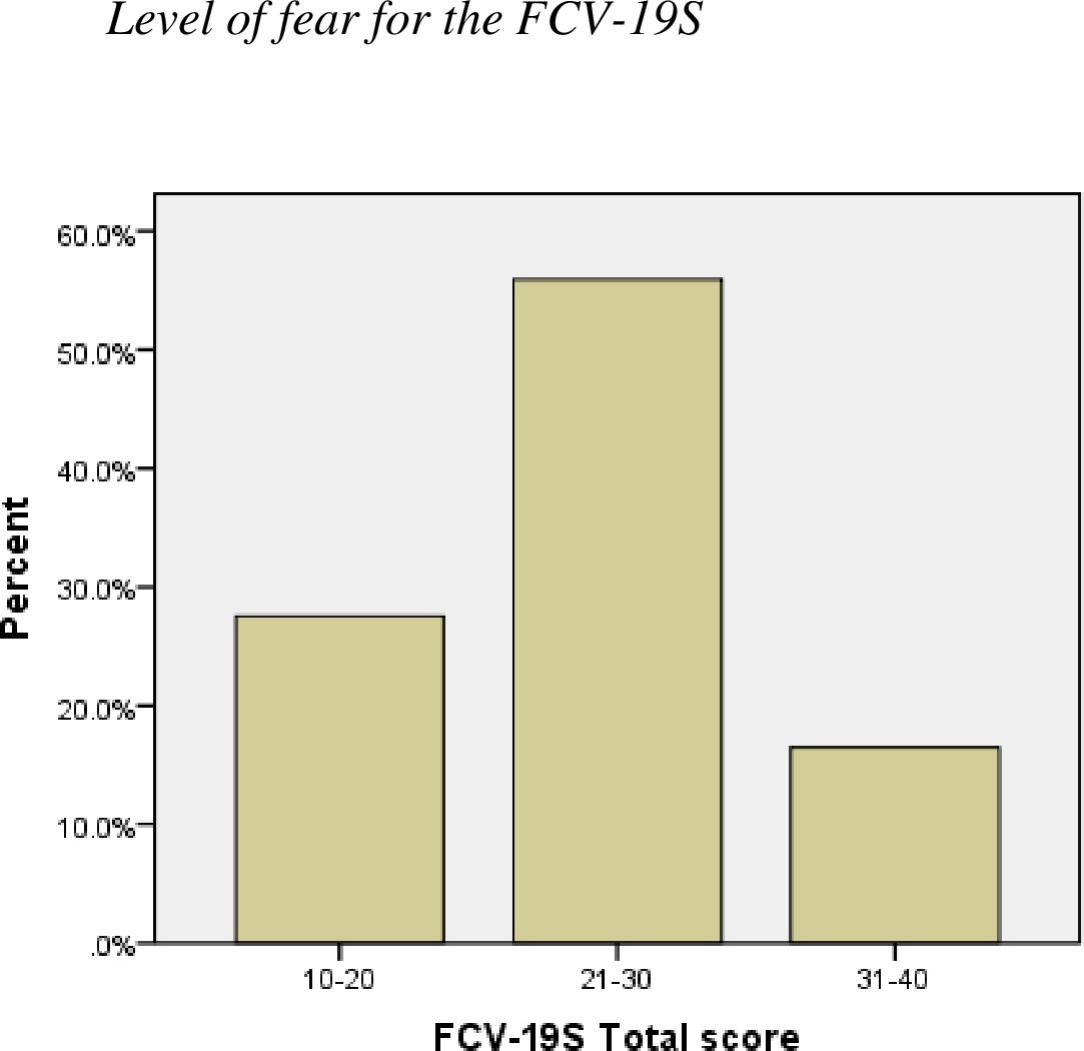
Level of fear for the FCV-19S.

Table 2 presents the results for the FCV-19S, which reflected the participants’ fear of COVID-19. For each item, the mean score exceeded the midpoint of 2.5, indicating a moderate level of fear. The total mean score for the FCV-19S was 23.64, (SD = 6.85) which again exceeded the mid-point for the total score range (21), indicating elevated level fear of the COVID-19 pandemic. Fig 1 shows the distribution of the fear level scores. Most participants 55% fear level was between 21–30 and 15% between 31–40 (Fig 1).

**Table 2:** Assessment of fear, anxiety, depression, stress, and perceived social support, (N = 365).

No.	Scale	Mean	SD
**FCV-19S**			
	FCV-19S total score	23.64	6.85
**DASS**			
1	Depression	21.30*	10.86
2	Anxiety	20.37**	10.80
3	Stress	23.33***	10.87
**MSPSS**			
1	Total score for significant Other Subscale	5.17	1.28
2	Total score for family Subscale	5.12	1.50
3	Total score for friends Subscale	5.09	1.18
	Total score for the scale	5.09	1.18

SD: standard deviation.

FCV-19S: Fear of COVID-19 Scale.

DASS scoring.

Depression: Normal 0–9, mild, 10–13, moderate 14–20

*severe 21–27, extremely sever 28+

Anxiety scoring: Normal 0–7, mild, 8–9, moderate 10–14, severe 15–19

**extremely sever 20+

Stress scoring: Normal 0–14, mild, 15–18

***moderate 19–25, severe 26–33, extremely sever 34+

MSPSS Multidimensional Scale of Perceived Social Support.

### Assessment of depression, anxiety, and stress

**Fig 2:**
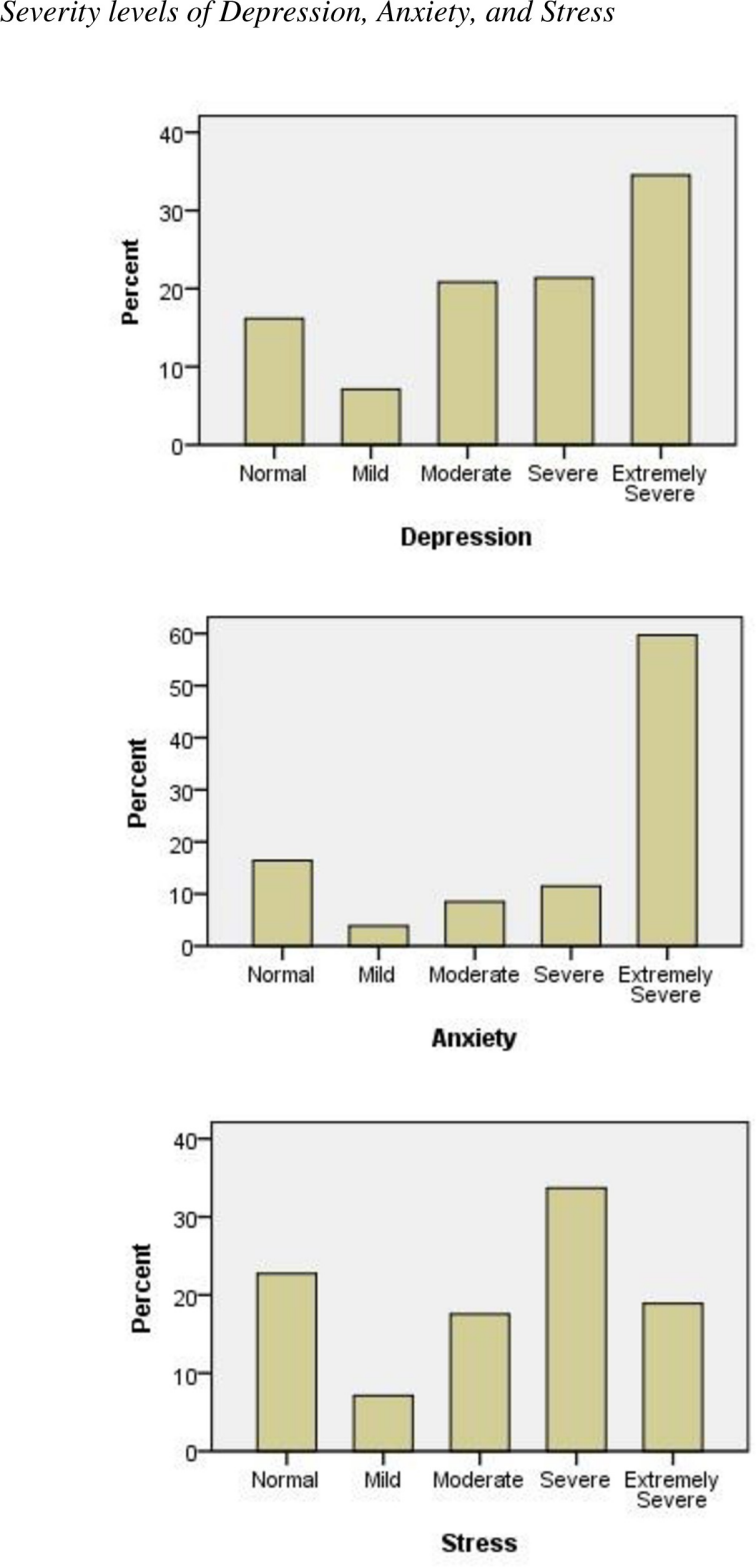
Severity levels of depression, anxiety and stress.

The mean scores for each subscale of the DASS, are presented in Table 2. Participants displayed extremely severe depression (21.30 ± 10.86), extremely severe anxiety (20.37 ± 10.80), and moderate stress (23.33 ± 10.87). Fig 2 illustrates, for depression, anxiety, and stress, the distribution of the participants across the five levels of severity. Based on the data, approximately 35% of the participants had extremely severe depression, over 40% had moderate to severe depression, and approximately 20% had normal to mild depression (Fig 2). For anxiety, approximately 60% of the participants, reported extremely severe anxiety. Regarding stress, the figure shows an uneven distribution over the severity levels, indicating inconsistent patterns of stress severity. However, approximately 35% was severely distressed.

### Perceived social support

The results regarding the social support received by the health-care workers from significant others, family members, and friends, respectively, are presented in Table 2. For significant others, the results indicated that the participants perceived high levels of support from all associated sources; the scores for all items exceeded 5 out of 7. These high scores were reflected in the mean score for the subscale (5.17 out of 7), which exceeded the midpoint. Regarding the family subscale, for all associated items the mean scores were above the midpoint of 4, indicating adequate support from family members. The mean score for the subscale (5.03 out of 7) was also above the midpoint, indicating high recognition of family support. Similarly, for the friends subscale, for all items the mean scores were above the midpoint, and the mean score for the subscale (5.05 out of 7) indicated high recognition of support from friends. The total mean score for the MSPSS was 5.09 out of 7, indicating high perceived social support (Table 2).

**Fig 3:**
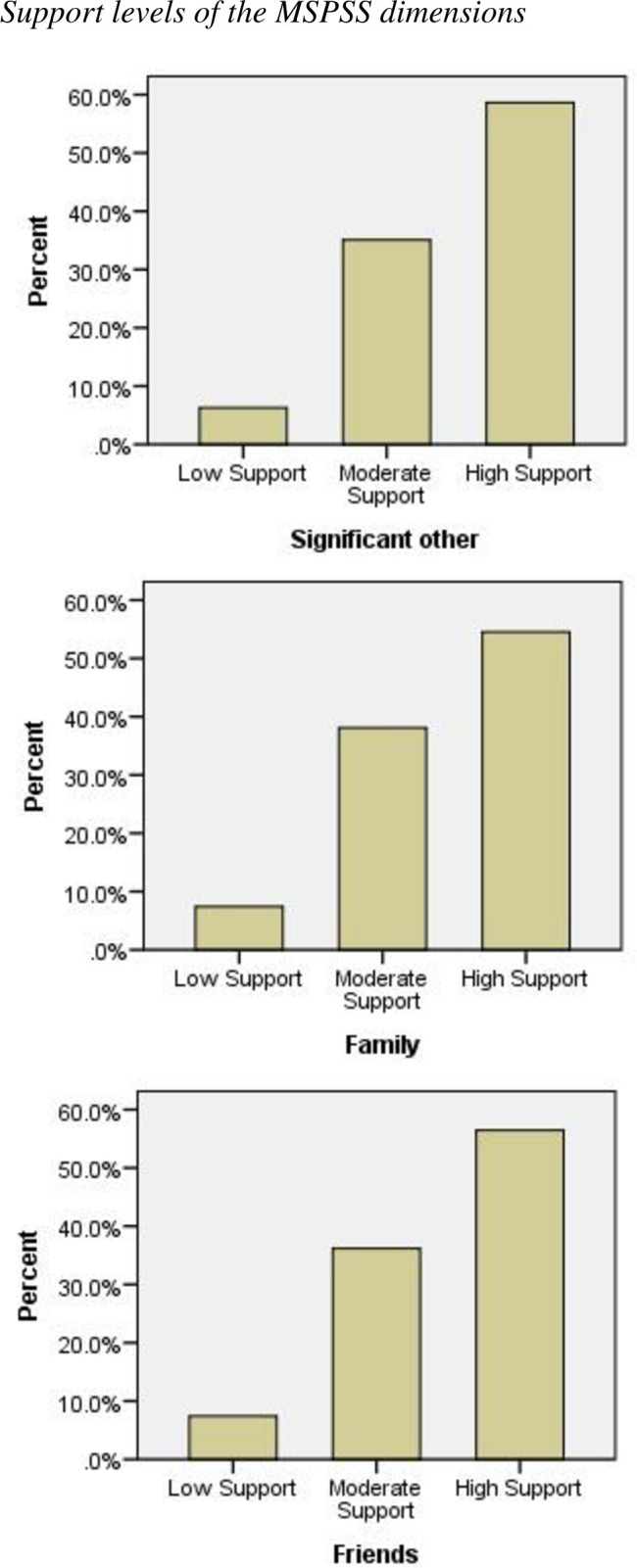
Support levels of the MSPSS dimensions.

Fig 3 shows the distribution of the scores for the three dimensions over three levels of support (low, moderate, and high support, respectively). The figure shows that all three dimensions are consistently distributed across the three levels. The highest frequency was reported for “high support,” followed by “moderate support,” and “low support,” respectively. This pattern was consistent across all three dimensions (Fig 3).

### Variations across demographic sub-groups

The main differences between the demographic sub-groups in terms of the study variables (Table 3) (fear, anxiety, depression, stress, and perceived social support) are listed below:

- Male participants returned statistically higher scores for fear, depression, anxiety, and stress, respectively, when compared to female participants (p < 0.001, p = 0.001, p < 0.001, and p = 0.001, respectively). However, no statistical difference was found between males and females regarding social support.
- Married participants returned significantly higher scores for fear, depression, anxiety, and stress, respectively, when compared to single participants (p = 0.015, p = 0.004, p = 0.019, and p = 0.012, respectively). In addition, married participants demonstrated higher social support when compared to single participants (p < 0.001).
- Participants aged over 40 years showed statistically higher levels of fear, depression, anxiety, and stress, respectively, when compared with participants aged < 40 years (p < 0.001, p < 0.001, p = 0.001, and p < 0.001, respectively). Moreover, older participants (> 40 years old) showed higher perceived social support than younger participants (< 40 years old; p = 0.001). The result of ANOVA (Table 4) revealed that significant relationship between psychological distress and social support and age *p* ≤ 0.05.
- Similarly, participants with more clinical experience (over 20 years) showed statistically higher levels of fear, depression, anxiety, and stress, respectively, when compared to participants with clinical experience of less than 20 years (p < 0.001, p < 0.001, p < 0.001, p < 0.001, respectively). Further, participants with more clinical experience reported more social support when compared to participants with shorter clinical experience (p = 0.018). The result of ANOVA (Table 5) revealed that significant relationship between psychological distress and social support and clinical experience, p ≤ 0.05.

- Participants who provided care for patients who had tested positive for COVID-19 reported higher levels of fear, depression, anxiety, and stress, respectively, when compared to those who did not provide care for patients who were COVID-19-positive (p < 0.001, p < 0.001, p = 0.002, p = 0.001, respectively).
- Participants who took vacation days during the pandemic reported lower levels of fear, depression, anxiety, and stress, respectively, than did those who did not take any vacation during that period (p = 0.000, p = 0.002, p = 0.000, p = 0.000, respectively). However, in relation to social support, there was no significant difference between the participants who took vacation days and those who did not take vacation days (p = 0.319).
- No significant differences were observed between professions (nurses, doctors, radiologists, and pharmacists) regarding any of the study variables (fear, depression, anxiety, stress, and social support).

**Table 3:** Independent t test results for sociodemographic variables regarding fear, anxiety, depression, stress, and perceived social support.

Outcome variables	Ind Variables	Mean ± SD	df	T value	P value
	**Gender**		363		
	**N = (Male 162.Female 202)**				
Fear	Male	25.07±6.68		3.644	0.000***
	female	22.49±6.78			
Depression	Male	23.44±10.07		3.418	0.001***
	Female	19.59±11.18			
Anxiety	Male	22.73±9.99		3.809	0.000***
	female	18.48±11.08			
Stress	Male	25.37±10.01		3.250	0.001***
	female	21.70±11.26			
Social support	Male	5.17±1.15		1.255	0.210
	Female	5.02±1.20			
	**Marital status**		363		
	**N = (married 210,single 155)**				
Fear	Married	24.38±6.79		2.436	0.015*
	Single	22.63±6.82			
depression	Married	22.69±10.68		2.874	0.004**
	Single	19.42±10.84			
Anxiety	Married	21.50±10.86		2.357	0.019*
	Single	18.83±10.56			
stress	Married	24.56±10.87		2.533	0.012*
	Single	21.66±10.66			
Social support	Married	5.28±1.04		3.683	0.000***
	Single	4.83±1.30			
	**Took care of patients diagnosed positive Corona virus**				
	**N = (Yes.136, No 229)**				
Fear	Yes	25.70±6.29		4.553	0.000***
	No	22.41±6.88			
depression	Yes	23.94±10.51		3.640	0.000***
	No	19.73±10.78			
Anxiety	Yes	22.63±10.63		3.124	0.000***
	No	19.02±10.70			
Stress	Yes	25.82±10.35		3.421	0.000***
	No	21.85±10.91			
Social support	Yes	5.08±1.18		0.041	0.967
	No	5.09±1.18			
	**Took vocation days during pandemic**				
	**N = (Yes.150, No 215)**				
Fear	Yes	21.77±5.59		4.6487	0.000***
	No	24.62±5.88			
depression	Yes	19.52±7.71		3.0158	0.002**
	No	22.15±8.52			
Anxiety	Yes	19.25±6.58		5.7175	0.000***
	No	23.31±6.74			
Stress	Yes	18.67±6.14		5.1074	0.000***
	No	22.36±7.21			
Social support	Yes	4.99±1.10		0.9974	0.319
	No	5.10±0.98			

* statistically significant at (α≤ 0.05)

** statistically significant at (α≤ 0.01)

*** statistically significant at (α≤ 0.001).

**Table 4:** One way ANOVA results for sociodemographic variables regarding to fear, anxiety, depression, stress, and perceived social support.

Outcome variables	Independent Variables		Mean ± SD	df	F value	P value
	N	Age group/years				
Fear	117	20–29	22.29±6.69	4,360	5.553	0.000**
	79	30–39	21.99±6.67			
	88	40–49	25.38±6.77			
	61	50–59	25.85±6.29			
	20	>60	23.60±7.50			
Depression	117	20–29	19.52±10.91		5.770	0.000**
	79	30–39	18.42±10.55			
	88	40–49	23.65±10.65			
	61	50–59	25.44±9.64			
	20	>60	20.15±11.21			
Anxiety	117	20–29	18.68±10.41		4.939	0.001**
	79	30–39	17.95±11.03			
	88	40–49	22.09±10.60			
	61	50–59	24.66±9.95			
	20	>60	19.15±11.47			
Stress	117	20–29	21.18±10.54		7.376	0.000**
	79	30–39	20.25±11.27			
	88	40–49	25.80±10.13			
	61	50–59	28.15±9.36			
	20	>60	22.50±11.97			
Social support	117	20–29	4.37±1.23		4.799	0.001**
	79	30–39	5.07±1.25			
	88	40–49	5.37±1.09			
	61	50–59	5.27±1.03			
	20	>60	5.36±0.92			

** statistically significant at (α≤ 0.001).

**Table 5:** One way ANOVA results for sociodemographic variables regarding to fear, anxiety, depression, stress, and perceived social support.

Outcome variables	Ind Variables		Mean ± SD	df	F value	P value
	N	experiences /years		2.362		
Fear	119	<10	22.55±6.54		9.939	0.000**
	152	10–19	22.85±7.02			
	94	>20	26.28±6.30			
Depression	119	<10	19.65±10.33		14.005	0.000**
	152	10–19	19.55±10.97			
	94	>20	26.23±9.92			
Anxiety	119	<10	18.76±10.17		12.190	0.000**
	152	10–19	18.78±11.05			
	94	>20	24.97±9.93			
Stress	119	<10	20.37±9.93		12.714	0.000**
	152	10–19	21.53±11.45			
	94	>20	21.82±9.66			
Social support	119	<10	4.84±1.14		4.077	0.018*
	152	10–19	5.16±1.25			
	94	>20	5.27±1.06			

* statistically significant at (α≤ 0.05)

** statistically significant at (α≤ 0.001).

### Factors influencing social support during the COVID-19 pandemic

According to the correlation matrix presented in Table 6, both clinical experience and social support have a weak significant positive correlation with fear, depression, anxiety, and stress, with correlation values (*r*) being approximately 0.20 and below. However, fear, depression, anxiety, and stress were positively correlated, with correlation values (*r*) ranging between 0.60 and 0.90; this indicated strong relationships.

**Table 6:** Inter-correlation matrix of variables associated with social support during the COVID-19 pandemic.

Variables						
	Clinical experience	Social support	Fear	Depression	Anxiety	Stress
**Clinical experience**	1					
**Social support**	.142**	1				
**Fear**	.198**	.170**	1			
**Depression**	.219**	.124*	.634**	1		
**Anxiety**	.208**	.144**	.657**	.935**	1	
**Stress**	.218**	.134*	.641**	.920**	.923**	1

Correlation included only infertile participants.

** Correlation is statistically significant (α = 0.01) (two-tailed).

* Correlation is statistically significant (α≤ 0.05) (two-tailed).

## Discussion

The findings of the present study provide insights into health-care workers’ psychological status during the COVID-19 Pandemic. This study analyzed a mixed group of health-care workers in Jordan five months after COVID-19 was declared a pandemic.

Factors associated with health-care workers’ psychological distress were determined to include being male, married, aged 40 years and older, having more clinical experience, and working directly with patients who have been diagnosed with COVID-19. Fear, depression, anxiety, and stress were positively correlated. All participants reported psychological distress; however, those who were 40 years of age and older showed a statistically higher level of psychological distress. The health-care workers’ concerns were due to several factors. A possible reason for the high level of distress among older workers is that the risk of severe respiratory distress as a result of COVID-19 increases with age, meaning older adults are at higher risk [33]. People at increased risk and those who live with or visit such people need to take precautions to protect themselves from getting COVID-19. Thus, older health-care workers may have reported higher psychological distress because older people can have health issues that make them more prone to complications, and they could also live with young children and/or have older people in their extended family, which could cause them to worry about bringing the virus home to their family members.

The current study’s findings also indicated that health-care workers who took vacation days reported lower levels of depression, fear, anxiety, and stress, respectively. These results support those of Luceno-Moreno et al. [34], who established that long working hours contribute to psychological problems, and those of Barello et al. [35], who observed work-related psychological pressure, emotional burnout, and somatic symptoms among health-care workers in Italy. The impact of working long shifts, 12 hours and more, on nurses and health-care assistance found 24% of nurses and health-care assistance were more likely to miss days of work due to sickness [36]. Thus, health-care workers are encouraged to take vacations from work for helping health-care workers relax, which contributes to preventing stress. Therefore, during pandemic situations vacations from work are necessary for reducing psychological distress among health-care workers, leading to lower levels of depression, fear, anxiety, and stress. Of course, the effectiveness of this can depend on the local quarantine policy and burden experienced by health-care workers.

The study results indicated weak correlations between years of clinical experience and fear, anxiety, and depression, respectively. The challenge that the pandemic brining to health-care workers such increase acuity of care and increased patients’ volume and, uncertainty health-care professional safety, as a result of reusing of personal protective equipment which was not part of health-care professional practice [37]. Health-care workers are hearing about potential surge, which was expected to hit harder, dealing with severe ill patients and death. With experience, health-care workers may adjust to stressful working environment but research, however, stressors may accumulate and cause psychological distress [38].

Pappa et al. [39] conducted a systematic review and meta-analysis of the prevalence of anxiety, depression, and insomnia, respectively, among health-care workers during the COVID-19 Pandemic. Anxiety was assessed across 12 studies, and a prevalence of 23.2% was returned; meanwhile, depression was assessed across 10 studies, and a prevalence of 22.8% was returned. The findings of Pappa et al. [39] support the results of the current study, as they indicate that health-care workers experience anxiety and depression during COVID-19; however, Pappa et al.’s findings also contradict the results of the present research, as the systematic review and meta-analysis showed a higher prevalence of anxiety than depression. Our study found higher depression than anxiety. Finally, Labrague and De Los Santos [40] found that 123 of 325 (37.8%) nurses examined had dysfunctional anxiety levels. Labrague and De Los Santos [40] also indicated that COVID-19 anxiety is associated with social support, organizational support, and personal resilience. These findings support the current study results by showing that front-line nurses are affected by anxiety during the COVID-19 Pandemic. To help health-care workers provide care under extremely difficult clinical circumstances such as COVID-19 pandemic, the emotional and behavioral reactions vary among health-care workers should be acknowledge and empowered through education and training to overcome fear and empathetic distress [37].

The results of our examination of social support during the COVID-19 Pandemic indicated that health-care workers perceive themselves as receiving high levels of social support. Our findings showed that health-care workers gain social support when providing care to patients. The health-care workers we examined perceived high levels of all types of social support, with mean item scores exceeding 5 out of 7 for all. The overall mean score (5.17 out of 7; determined by considering the means for all three subscales) was also above the midpoint. Thus, the results showed that, during the COVID-19 Pandemic, health-care workers gain support when providing care for patients. These results support the findings of a narrative review by Heath et al. [13], which showed that support offered before and during an incident influences whether health-care professionals experience injury or psychological growth. Heath et al. [13] also indicated that clinicians who have healthy, meaningful personal and professional relationships are contented and have a lower risk of burnout. Heath et al. also showed that health-care professionals who have work responsibilities that interfere with their home lives are more likely to experience burnout, leading to stress when providing care to patients [13]. Also, feeling the guilt of transmitting the infection to family members at home, health-care workers experience stigmatization. Self-stigma, mostly, if health-care workers were in direct contact with infected patients, they preferred to stay away from them [41]. Moral injuries have been widely reported among health-care workers on duty during the COVID-19 pandemic [10]. The clinical and ethical challenges that these workers face can foster psychological distress, and health-care workers with poor psychological health affect the quality of care provided at their institutions, as well as their coworkers capability to work [42]. Anticipating the problem may help lessen its impact, and early identification of psychological distress and health-care support is essential.

Indeed, COVID-19 infection becomes an occupational injury when health-care workers contract the virus through work or while commuting to work [11]. To support health-care workers during future health emergences (such as future infections or disasters) and protect them from such injuries, health-care leaders should, in accordance with other regulatory agencies around the world, rapidly implement policy changes at institutional levels and at the local level to facilitate a shift in culture towards improved well-being and workplace environments.

Social support is necessary as coping mechanism to decrease health-care workers’ psychological distress and promote positive feelings. Spinale et al. [43] reported that social support is correlated with spirituality. Spirituality is associated with transcendental values that are generally influenced by personal experiences and grounded in religious traditions; however, a comparable sentiment can be achieved in a non-religious context. Spirituality can foster positive feelings and promote physical and mental health [43]. People with greater spirituality have also been reported to experience higher levels of well-being [44]. Thus, improving spirituality among health-care workers during pandemics may help them relieve their physical and psychological distress, and also support coworkers, patients, and patients’ family members. This is especially important during pandemics, as these are times when spiritual specialists or religious leaders are unable to closely contact patients and health-care workers.

In summary, the present findings show that health-care workers feel depressed, anxious, stressed, and fearful of the pandemic. This means that health-care workers are making critical decisions in the course of their work while experiencing notable distress. Direct support from management can help staff develop positive perceptions about work, and can help them manage stress. However, inadequate protection, perceived stigma, and negative feedback from patients can exacerbate COVID-19-related psychological distress [45, 46]. Also, health-care workers who perceive high level of psychological distress, need psychological support [47]. Que et al. [45] suggested that psychosocial interventions should be provided in the early stages of pandemics for health-care workers who are at risk of experiencing psychological distress. According to our findings, adequate social support is essential for addressing stress, anxiety, and depression. However, additional research is required to explore the long-term effects of the COVID-19 Pandemic on psychological distress among health-care workers.

### Strengths and limitations

The strength of this study is that it measured psychological distress and social support among health-care workers five months after the WHO declared the COVID-19 outbreak a pandemic, and after public services in Jordan were reopened after the lockdown. The study also considered health-care workers’ psychological concerns after the pandemic was declared. This is a strength because psychological distress among health-care workers during the pandemic has been somewhat understudied. On the other hand, this study also contains limitations. One of the principal limitations is the cross-sectional nature of the study. Psychological distress was only evaluated cross-sectionally; consequently, we could not obtain information regarding existing causal relationships. Further, the data did not represent the entire population of health-care workers and the services in which they worked (intensive care, primary care. . .), also, did not include other variables such as whether the participants had had any personal experience of loss or illness due to COVID in their family or friends, and, as a result, the findings cannot be used to make useful generalizations regarding health-care workers as a whole, or to determine specific variables’ correlations with specific groups of health-care workers. A larger sample of health-care workers recruited from various areas in Jordan is needed to verify the results. Moreover, further research is needed to explore the long-term effects of the COVID-19 Pandemic on health-care workers.

### Implications for health-care workers

The results of this study showed that the COVID-19 Pandemic has fostered psychological distress among health-care workers in Jordan, and health-care workers have become acutely conscious of the threat of the virus’ spread. Thus, safeguarding the psychological well-being of health-care workers is crucial during pandemic situations. Employers should endeavor to identify approaches that can improve psychological distress among such workers.

Most health-care workers have direct contact with patients, and this can cause high levels of anxiety. Managers and leaders should increase the support available for health-care workers in their organizations and in health-care workers’ own social networks. Early identification of psychological stress is important.

Being male, older, and having more clinical experience increase the risk of stress during pandemics. Thus, during such situations psychological support is essential for this group. However, older health-care workers should also proactively seek psychological support. Further, efforts should be made to develop coworker support; health-care workers could aim to help others implement effective decision-making in response to pandemic-related challenges.

### Practical implications

The results of this study suggest that measures should be implemented to protect the mental well-being of health-care workers during the COVID-19 Pandemic. Leaders in health-care facilities should realize the importance of close relationships with health-care workers during the extraordinary times they are facing in this pandemic. In addition to ensuring that the physiological needs of health-care workers, such as availability of PPE and safe working environments, are met, leaders should reassure health-care workers that they and their families will be adequately supported should they become infected with COVID-19. This support should include medical, financial, and psychosocial assistance for both the health-care workers and their families. Moreover, leaders and managers of health-care facilities should make efforts to identify sources of anxiety and fear among health-care workers, and should schedule rigorous assessments by professional psychologists and mental-health professionals. At the primary and secondary levels, regular meetings should be held with health-care workers to promote the development of healthy patterns of coping with the stressors of working with patients with or suspected of having COVID-19. At the secondary level of prevention, individual counseling for mental well-being concerns and early treatment is essential. Teams of professional psychologists should be available at each institution for health-care workers to contact at any time, and prompt treatment should be provided, and as follow-ups. In addition, peer support and group discussions should be encouraged.

The major issues for health-care workers are fear, depression, anxiety, and stress. The participants in this study felt that they received high social support, but they also showed higher psychological distress. These characteristics should be considered when developing strategies to address this. It is not clear whether health-care workers physically distance themselves from their families as a result of lockdowns, social-distancing recommendations, and their close contact with patients. If so, social support in the workplace could give health-care workers a sense of being a member of a social network; consequently, health-care workers should be provided with opportunities to establish and strengthen such professional relationships.

Further, healing moral distress and occupational injuries are important. This requires collaboration between health-care workers, administrators, and representatives of the community; in particular, an ethically admissible code for pandemic contexts should be established that can strengthen health-care workers’ morals.

## Conclusion

Our study demonstrated the presence of fear, depression, anxiety, and stress among health-care workers during the COVID-19 Pandemic. The health-care workers examined considered social support from families and friends to be important during the pandemic, and demonstrated a need for increased social support to adjust to psychological distress. Factors determined to be associated with psychological distress were being male, married, aged 40 years or older, and having more clinical experience. The influence of these factors may be related to the environment in which health-care workers practice. Thus, this study suggests that health-care organizations pay attention to health-care workers’ well-being and promote early assessment and identification of psychological distress. It is also necessary to address social support through policy since, as a result of social distancing, there are fewer opportunities for social interaction and to attend events. Social support systems play an important role in protecting health-care workers and reducing the prevalence of psychological distress.

## Supporting information

S1 File (XLSX)
